# A longitudinal Q-study to assess changes in students’ perceptions at the time of pandemic

**DOI:** 10.1038/s41598-023-36003-9

**Published:** 2023-05-30

**Authors:** Noori Akhtar-Danesh, Danielle Brewer-Deluce, Jessica Saini, Sarah Wojkowski, Ilana Bayer, Anthony N. Saraco, Courtney Pitt, Bruce Wainman

**Affiliations:** 1 School of Nursing, McMaster University, Hamilton, ON Canada; 2 Education Program in Anatomy, McMaster University, Hamilton, ON Canada; 3 Department of Pathology and Molecular Medicine, McMaster University, Hamilton, ON Canada; 4 School of Rehabilitation Science, McMaster University, Hamilton, ON Canada; 5 School of Interdisciplinary Studies, University of British Columbia, Vancouver, BC Canada

**Keywords:** Anatomy, Medical research, Mathematics and computing

## Abstract

The COVID-19 pandemic forced many universities and colleges to rapidly adopt online course delivery. As with any new foray, realizing the optimal aspects of a course to change became incredibly important for course instructors. In this study, we used a particularly sensitive method, i.e. Q-methodology, to evaluate changes based on students’ perceptions from fall 2020 to winter 2021. Q-methodology is commonly used to uncover shared values, opinions, and preferences. Using Q-methodology, students participating in both semesters of an undergraduate anatomy and physiology course were surveyed in fall 2020 and winter 2021. The Q-sample included 44 statements. Data from fall 2020 were treated as the baseline and changes in students’ perceptions from 2020 to 2021 were assessed. In total, 31 students completed both fall 2020 and winter 2021 course evaluations. Three salient factors emerged from the fall 2020 evaluation: Overtaxed students, Solo Achievers, and In-Person Learners. At the baseline, students were concerned mostly about the delivery of the course, then the winter 2021 evaluation showed how they were adjusting to online learning. The longitudinal Q-study proved to be robust in identifying changes in perceptions. These granular findings indicate how students might differ in viewing and evaluating online courses. This methodology can be used in redesigning and restructuring different components of an online course in higher education settings.

## Introduction

In early 2020 the COVID-19 pandemic restricted universities and colleges worldwide to have limited access to in-person education and forced them to adopt online course delivery^1–3^. This rapid switch to online delivery complicated course delivery in unique ways. For instance, anatomy education has unique barriers for online teaching^2,4^ where, historically, physical cadaveric specimens have been used as the primary method of teaching^5,6^. Currently, in Canada all undergraduate medical programs use cadavers for teaching anatomy^7^.

This consistency in the use of cadavers in anatomy teaching may be due to the ability of physical specimens in teaching visuospatial concepts as one of the most demanding aspects of anatomy education^6,8^. Educators also cite tacit benefits to cadaver use; namely, developing skills within a “hidden curriculum” regarding ethics and professionalism^4,6,9,10^. However, learning with cadavers necessitated proximity which were not compatible with the COVID-19 social distancing restrictions^6^. In addition, students were no longer able to have access to accompanying aids, such as models, pathology specimens, and skeletons which are normally found in the anatomy laboratory^9^.

These barriers and changes in the course delivery approach have greatly affected students and how they may have coped, viewed, and evaluated their courses and the online delivery methods. For example, studying perception of students of an online histology course showed that most students did not have technical problems, but those who did, the experience was quite frustrating^3^. Indeed, the online anatomy education was discussed in a special issue of Anatomical Sciences Education in 2020 (Vol. 13, Issue 3).

On the other hand, the switch to online education presented a unique opportunity to perform critical course evaluations to supply the instructors with rapid feedback about the changes to be made, and ultimately, whether they were effective. To conduct such a study, we used a Q-methodology design for data collection and analysis. Although Q-methodology has recently been used for end-of-term course evaluation and course improvement^11,12^, the use of Q-methodology for longitudinal course evaluation is novel.

In this study we evaluated students’ perceptions regarding an undergraduate anatomy and physiology course twice: once in the fall 2020 semester when we were less than 1 year into the pandemic, and then again in the winter 2021 semester when we had learned much efficient use of technology for teaching and student evaluations. To obtain sufficiently granular and prioritized data, we used a longitudinal Q-study design and investigated the changes in students’ attitudes from fall 2020 to winter 2021. The main objectives were to understand the students’ perceptions about the introductory anatomy and physiology course in the fall 2020; and if and how it changed in the winter 2021.

## Methods

In this section, first a brief review of a Q-methodology study is provided. Then, different steps of this study are presented based on a Q-methodology study framework.

### Q-methodology

**Figure 1:**
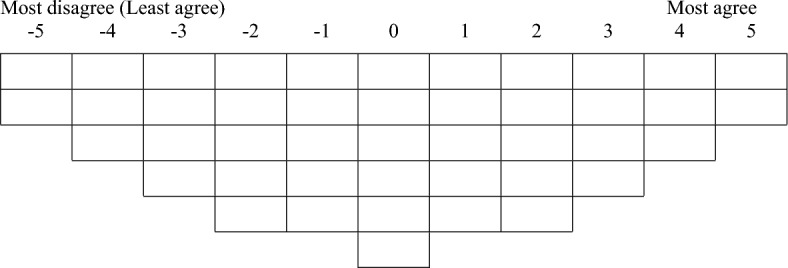
Q-sort table with 44 cells which is equal to number of statements in the Q-sample.

Q-methodology is a combination of qualitative and quantitative methods introduced in 1935 by William Stephenson^13,14^. In a Q-methodology study, the main objective usually is to identify patterns of thought instead of the numerical distribution of different groups among participants. It is a useful methodology in exploring human perceptions and interpersonal relationships by identifying similarities and differences in perceptions between groups^15^. Q-methodology allows for systematic examination and greater understanding of the connections between subjective statements^16,17^. To conduct a Q-study, a representative list of statements, known as a Q-sample, is needed for data collection. This set of statements can be assembled from literature, previous Q-studies, or by collecting statements from potential study participants. After identifying the statements in the Q-sample, a Q-sort table (grid) with quasi-normal distributions is developed for data collection (for example see Fig. 1). This table has as many cells as the number of statements in the Q-sample. The Q-sort table includes a rating scale across the top that can range, say, from – 3 to + 3 to – 6 to + 6. This range usually depends on the number of statements in the Q-sample so that a larger number of statements requires a wider range. However, the results of a Q-methodology study are quite robust with respect to the range and distribution of the Q-sort table^17^, therefore, both the range and distribution can be altered for the convenience of the participants. The Q-sort table is used for data collection and each completed Q-sort table is known as a Q-sort.

Additionally, because the objective in Q-methodology is to identify the range of opinions, not their distributions, in the study participants the sample size is not a determining factor and Q-studies usually use small sample sizes and low response rates do not bias the study results^18^. As mentioned above, a Q-methodology study comprises qualitative and quantitative components. The quantitative component includes a by-person factor analysis of Q-sorts to classify (factorize) participants into different groups, so that each factor includes participants with similar views or perceptions regarding the topic of the study.

Next, each factor is usually described based on a unique set of statements called *distinguishing statements*. A distinguishing statement for each factor is a statement with a score for the factor that is significantly different from its factor scores on all the other factors^16^. Finally, for statistical analysis we used the QPAIR program in Stata which is currently the only program available for systematic analysis of paired Q-sorts^19,20^. A glossary of terms used in Q-methodology is provided in Supplementary Table S1.

The face validity of the statements used in a Q-methodology study can be assessed by using the exact wording of the statements from participants and the literature, although the statements can be slightly edited for grammar and readability^21^. The content validity of statements is assessed by domain experts^21,22^. With regard to the validity of the Q-sorting operation, it provides an opportunity for the participants to express their inner subjective views, and there is no external criterion to evaluate or judge an individual’s response or feeling to a statement^18^. As a result, participants’ completed Q-sorts are regarded as valid expressions of their perceptions. Test–retest reliability of Q-sorting process has been reported to be quite high (≥ 0.80) in several studies^17,23,24^.

### Q-sample (sample of statements)

In this study the Q-sample was developed from the literature, previous studies, as well as students’ feedback from the annual, university-mandated course evaluations about their experiences with the course content and delivery at the time of pandemic. The statements were then reviewed by three senior members of the research team and four students for content validity, face-validity, and readability. The final Q-sample included 44 statements (Supplementary Table S2). Further theoretical information regarding Q-methodology can be found in Brown^17^ and a step-by-step procedure is provided in Akhtar-Danesh et al.^16^ and Brewer-Deluce et al.^11^.

### Participants (Q-set)

The participants in a Q-study are usually known as Q-set^25^ in contrast with Q-sample which refers to the statements used in the study for data collection. The students enrolled in the anatomy course included approximately 55% Bachelor of Health Sciences (BHSC) students, 30% Integrated Biomedical Engineering and Health Sciences (iBioMed) students, 10% Midwifery students, and 5% Engineering students. This two time-point, case study only includes students who participated in both evaluations.

### The Q-sort table and data collection

For the 44 statements included in the Q-sample, we developed a table with 11 columns and anchored with − 5 (most disagree or least agree) at the left and + 5 (most agree) at the right end of the table (Fig. 1). The columns were sequentially numbered from left (− 5) to right (+ 5). Then, the Q-sort table, the statements, and an instruction sheet were presented to each student in fall 2020 before the midterm exam. The instruction sheet included a detailed instruction on how to complete the Q-sort table. In addition to completing the Q-sort table, the students were asked to provide open-ended statements to explain their rationale for each statement that they rated as strongly agree (+ 5) or strongly disagree (− 5). The students also completed a demographic questionnaire which included questions regarding gender, age, and educational background.

### Statistical analysis

We assessed change in perception from 2020 to 2021 as the outcome of interest. We used the free QPAIR program in Stata^20^ for analysis, and assumed the fall 2020 evaluation as the baseline. First, we used a by-person factor analysis with principal component factor extraction and varimax rotation to identify the factors from fall 2020. Factor scores were identified and a Cohen effect size of 0.80 was used for identifying distinguishing statements for each factor^26,27^. Next, each factor was named based on its distinguishing statements. Then, for each factor (i.e. each group of students) the mean scores of the distinguishing statements from the second evaluation were compared with the factor scores from the first evaluation and the significant differences from Time 1 to Time 2 were identified. In addition to identifying distinguishing statements for each factor, the analysis provides a set of *consensus statements* where all participants agree/ disagree at the same level with each statement. The details of factor score calculations are fully explained in Akhtar-Danesh and Wingreen^19^.

### Ethics declaration

The study protocol was approved by the Hamilton Integrated Research Ethics board (HiREB# 11355). All methods were performed in accordance with relevant guidelines and regulations and all participants (i.e. students) provided written informed consent.

## Results

Overall, 31 students completed both fall 2020 and winter 2021 evaluations. Participants’ mean age was 20.0 years (SD = 3.2, min = 19, and max = 34). Three salient factors emerged from the fall 2020 evaluation, which included 29 students, 22 females (75.9%) and 7 males (24.1%) (Table 1). Two students did not load significantly on any factor. The students who contributed to the three salient factors were from the following programs: BHSc (15 students), iBioMed (9 students), Midwifery (4 students), and Engineering (one student). Further descriptions of each factor with its distinguishing statements and the significant changes in each factor from fall 2020 to winter 2021, as well as the consensus statements are presented below.

**Table 1:** Demographic profile (n (%)), based on the factor.

Demographic variable	Factor 1	Factor 2	Factor 3	Total
Sex				
Female	11 (91.7)	8 (66.7)	3 (60.0)	22 (75.9)
Male	1 (8.3)	4 (33.30	2 (40.0)	7 (24.1)
Program				
BHSc	8 (66.7)	3 (25.0)	4 (80.0)	15 (51.7)
iBioMed	3 (25.0)	6 (50.0)	0 (0.0)	9 (31.0)
Midwifery	0 (0.0)	3 (25.0)	0 (0.0)	4 (13.8)
Engineering	1 (8.3)	0 (0.0)	0 (0.0)	1 (3.4)
Year of study				
1st	0 (0.0)	3 (25.0)	1 (20.0)	4 (13.8)
2nd	12 (100.0)	9 (75.0)	4 (80.0)	25 (86.2)
Total	12	12	5	29

*BHSc* bachelor of health sciences program, *iBioMed* integrated biomedical engineering and health sciences.

### Factor 1: overtaxed students

Twelve students loaded on this factor, all in their second year of studies. Eleven were female and one male. Eight students were from the BHSc program, one was from Engineering, and three were from the iBioMed program (Table 1). Table 2 presents their distinguishing statements at baseline (Time 1) and the average score for each distinguishing statement in winter 2021 (Time 2). At baseline, these students felt most strongly they needed more time to complete their multiple-choice question (MCQ) exam (+ 5); however, in winter 2021 they were neutral about this statement (0). In time 1, they were supportive of the statement “There needs to be more consistency between thee slides of the different lectures” (2) but became neutral to this statement in time 2 (0). Also, they were quite uncomfortable with the technology for studying anatomy online in the fall (Statement #5; − 2) but became supportive of online technology in the winter (+ 2). The other two statements that students’ perceptions changed markedly about were statement #37 “I think the lab modules are easy to follow”, where their score changed from − 3 at time 1 to 0 at time 2; and statement #20 “I believe having multiple professors from different areas of specialty is a strength of this course” where the score was − 4 in time 1 and changed to − 1 in time 2. Additionally, at time 1 the students indicated, “I feel like I am teaching myself. It is like paying tuition to watch YouTube videos” (+ 5) and they remained quite supportive to this statement at time 2 (+ 3).

**Table 2:** Distinguishing statements for overtaxed students, measured twice, at midterm (fall 2020- time 1) and at the end of term (winter 2021-time 2), during the anatomy and physiology course.

**#**	Statement	Factor score	
		Time 1	Time 2
18	I need more time to complete my MCQ exam	5	0*
25	I feel like I am teaching myself. It is like paying tuition to watch YouTube videos	5	3*
39	I think that the quality of teaching is worse than prior to the pandemic	3	2
15	I feel that the MCQ evaluations often require far more integration and application than we are taught in lecture, lab, and tutorial	2	1
17	There needs to be more consistency between the slides of the different lecturers	2	0*
43	I get confused because course information is on more than one platform	0	− 2
27	I think that the lectures fostered connections between anatomy and physiology	0	1
24	I have often found that the content in the lectures and the tutorials do not line up	− 1	− 2
5	I'm comfortable with the technology skills required for studying anatomy online	− 2	2*
37	I think the lab modules are easy to follow	− 3	0*
20	I believe having multiple professors from different areas of specialty is a strength of this course	− 4	− 1*
4	I think lectures covered an appropriate amount of content	− 5	− 4

Factor scores ranged from – 5 to + 5 and negative scores indicate disagreement.

Asterisk (*) indicates significant difference from time 1 to time 2.

### Factor 2: solo achievers

Twelve students loaded on this factor including 8 females and 4 males. Of this group, 3 were first-year and 9 were second-year students. Also, 3 students were from BHSc program, 6 from iBioMed program, and 3 from the Midwifery program. The distinguishing statements for this group are shown in Table 3. There were stark differences in this group’s attitude from fall 2020 to winter 2021. Although they strongly felt tutorial was useless to their learning (+ 4) at Time 1, their position changed to neutral in Time 2 (0). Also, at time 1 they scored the statement “I need more time to complete my MCQ exam” as neutral (0), but strongly disagreed with it in time 2 (− 4). The other statement they opposed at time 1 was “I think TA office hours are very helpful” (− 3), but they became agreeable to this statement in winter 2021 (+ 1). There was not much change in their attitude regarding the other distinguishing statements from Time 1 to Time 2.

**Table 3:** Distinguishing statements for solo achievers measured twice, at midterm (fall 2020- time 1) and at the end of term (winter 2021-time 2), during the anatomy and physiology course.

**#**	Statement	Factor score	
		Time 1	Time 2
8	Tutorials are useless to me	4	0*
18	I need more time to complete my MCQ exam	0	− 4*
15	I feel that the MCQ evaluations often require far more integration and application than we are taught in lecture, lab, and tutorial	− 2	− 3
2	I believe working in a group for peer teaches/presentations helped me learn and apply communication skills	− 2	− 2
42	I think creating peer teaches/presentations was a useful way to learn and remember content	− 3	− 2
30	I think TA office hours are very helpful	− 3	1*
32	Synchronous lab sessions were critical to my understanding of anatomy	− 5	− 4

Factor scores ranged from − 5 to + 5 and negative scores indicate disagreement.

Asterisk (*) indicates significant difference from time 1 to time 2.

### Factor 3: in-person learners

Five students loaded on this factor: three females and two males; one was a first-year student and four were second-year students. Four students were from the BHSc program and one from midwifery program. In general, there was no significant change in this group’s perceptions from Time 1 to Time 2 (Table 4). Specifically, they were in favor of in-person lectures. They had the highest score (+ 5) for the statement “Watching in-person lecturers use their body to emphasize concepts really helps cement them in my brain”, compared with scores of + 1 and + 2 that Factors 1 and 2 gave to this statement, respectively. However, their mean score slightly decreased for this statement in the winter (+ 3). Their highly negative scores for statements 35, 28, 18, and 15 were in contrast with the other two factors who either supported these statements (Factor 1) or were almost neutral (Factor 2). Interestingly, their attitude did not change toward these statements in Time 2.

**Table 4:** Distinguishing statements for in-person learners measured twice, at midterm (fall 2020- time 1) and at the end of term (winter 2021-time 2), during the anatomy and physiology course.

#	Statement	Factor score	
		Time 1	Time 2
41	Watching in-person lecturers use their body to emphasize concepts really helps cement them in my brain	5	3
9	I found it difficult to keep up throughout the semester—it was super easy to fall behind	1	0
33	I learned about the systems that work together in a holistic approach, rather than about individual, specific anatomy	1	3
11	The long answer worksheets are beneficial to my learning	− 1	− 2
35	I think we were tested too much on small insignificant names and details instead of bigger ideas	− 3	− 2
28	I think the way in which we are evaluated does not fairly represent what the material covered	− 3	− 3
18	I need more time to complete my MCQ exam	− 3	− 3
15	I feel that the MCQ evaluations often require far more integration and application than we are taught in lecture, lab, and tutorial	− 4	− 4

Factor scores ranged from − 5 to + 5 and negative scores indicate disagreement.

### Consensus statements

Table 5 includes the consensus statements at baseline and how the scores changed at the follow-up evaluation. Although there seem to be some changes, these statements mostly remained as the *consensus statements*. For instance, there was much higher agreement on statement #10 “I think that virtual specimens do not replace the physical presence of specimens” at time 2, indicating that although some students might have acclimatized to the virtual and digital world during the pandemic, they indicated that virtual specimens could not replace the physical presence of specimens for learning anatomy and physiology.

**Table 5:** Consensus statements for all students measured twice, at midterm (fall 2020- time 1) and at the end of term (winter 2021-time 2), during the anatomy and physiology course.

#	Statement	Factor 1	Factor 2	Factor 3
6	I find that there is not much distinction between synchronous labs and tutorials	1 (-2)	2 (4)	4 (2)
10	I think that virtual specimens do not replace the physical presence of specimens	2 (4)	5 (5)	4 (5)
44	I have difficulty understanding and practicing for the bellringer using the virtual specimens	3 (3)	4 (0)	3 (1)
23	I like that the asynchronous lectures allow me to stop, rewind, and listen to lectures multiple times	2 (4)	4 (4)	3 (4)
12	I think there should be transcripts for asynchronous lectures	4 (5)	2 (1)	2 (1)
14	I think there should be a standard set of slides/specimens that all groups will cover in synchronous labs and tutorials	1 (3)	3 (3)	2 (1)
21	I believe the transition to online school has removed the opportunity to learn from and communicate with other students	1 (3)	0 (2)	0 (3)
1	I feel that the expectations for the peer teachers/presentations are unclear	− 1 (− 3)	0 (− 2)	− 1 (− 1)
34	I think I would perform better on an in-person exam than an online exam	− 1 (0)	− 1 (− 1)	− 2 (− 2)
3	Asynchronous lab modules were critical to my understanding of anatomy	− 4 (− 4)	− 2 (− 2)	− 2 (− 3)
31	I prefer online learning compared to the in-person format	− 5 (− 5)	− 3 (− 2)	− 3 (− 5)
16	I find the synchronous sessions to be a toxic environment because some students will try to show off	− 3 (− 4)	− 4 (− 5)	− 4 (− 5)
7	Watching lectures was a waste of my time	− 3 (− 5)	− 5 (− 5)	− 5 (− 4)

Score ranges from − 5 to + 5 and negative scores indicate disagreement.

All students in the three groups were unanimous in their strong disagreement in Time 1 and Time 2 with statement #31 “I prefer online learning compared to the in-person format” and statement #7 “Watching lectures was a waste of my time”.

## Discussion

In this study, we used a novel approach for the assessment of change in perception using a paired Q-methodology design. We identified three groups of students (Overtaxed students, Solo Achievers, and In-person Learners), with distinctive point-of-views in fall 2020, which aligns with the beginning of pandemic, and assessed the changes in their viewpoints about the delivery and content of an online anatomy and physiology course from fall 2020 to winter 2021.

Q-methodology has been shown to be a useful approach to end-of-term course evaluations for examining students’ perceptions regarding course content and delivery^11,12,28^. Although literature is scarce in the use of this robust approach for evaluating change over time, our results demonstrate that Q-methodology can also reveal clear granular findings on changes in perceptions over time related to course evaluations.

At baseline, the *Overtaxed Students* were most concerned with having enough time for completing their MCQ exams (+ 5), felt as if they were paying tuition fees for watching videos, like YouTube, to teach themselves (+ 5). They also most strongly disagreed with the statements that “lectures covered an appropriate amount of content” (Statement #4, − 5), and they did not believe having multiple professors from different areas of specialty was a strength of the course (− 4). These granular findings may not be solely due to the rapid move to online delivery of the course, but also due to the stress imposed by the pandemic. Guldager et al.^29^ found 39% of students reported academic stress due to COVID-19, of which one third were concerned about their ability to complete the academic year, and one main factor associated with academic stress was female sex. Interestingly, eleven (91.7%) of our Overtaxed group were female students. O’Byrne et al.^30^ also reported a strong association between being female and stress among Danish health and medical science students during the COVID-19 pandemic. Nevertheless, except for statement #4 for which there was not much change in students’ altitudes from Time 1 to Time 2, there were considerable positive changes in the other statements which indicate their adaptability to the virtual learning environment.

In contrast with the other two groups, the *Solo Achievers* were disapproving of the tutorial aspects of the course. Initially, they felt strongly that tutorials were useless (Statement #8, + 4) and disagreed that teaching assistant (TA) office hours were helpful (Statement #30, − 3). They highly disagreed that synchronous lab sessions were critical to their understanding of anatomy (Statement #32, − 5). This might be indicative of the self-sufficiency and independency of this group of students. These findings resemble the findings by Pilkington & Hanif^31^ where students found asynchronous sessions more suitable than synchronous sessions for their learning. They also found low attendance rates of 20–25% for tutorial sessions. However, we found the students’ attitudes changed positively at Time 2 for Statements #8 and #30, respectively, but remained to be highly negative on Statement #32 (− 4). In addition, their position changed from neutral (0) to negative (− 4) on Statement #18 “I need more time to complete my MCQ exam”.

*In-person Learners* were skeptical about online learning at both Time 1 and Time 2. Their skepticism might be generated because of a lack of a sense of community and/or feelings of isolation, having technical issues and difficulties in collaborating with peers, the need for more disciplined learning environment, and lack of self-motivation^32^.

Although students’ attitudes changed positively toward online learning during the pandemic, which is in keeping with the findings of a recent study^32^, students still viewed the online classroom as suboptimal to in-person learning. However, compared to *In-person Learners*, who comprised a small group (17.2%) of students, *Overtaxed Students* and *Solo Achievers*, became more adaptable to online learning from Time 1 to Time 2 and developed more positive views regarding online learning.

The finding that students in general did not wish to learn in an online format and yet found lectures absolutely key to learning (Statement #7) undoubtedly set up a conflict between the necessity of the lecture material and distaste for the form of delivery. These findings confirm the transition from in-person to online learning is gradual, and students need time to become familiar with and adapt to online learning^33,34^, although it does not make online learning the preferred method of delivery. This finding is supported by the consensus statement #31 “I prefer online learning compared to the in-person format” that was strongly disagreed with by all students even though they all appreciated the opportunity of being able to review online lectures asynchronously (Statement #23). The reason students preferred the in-person classroom over online learning might be related to the notion that the pandemic forced an abrupt move to online learning that did not meet student expectations. As such, at the beginning of the course, students may have felt less engaged in the classroom and more distracted by the technical challenges related to an online format as they were adjusting to this style of learning^34–36^. The source of the dislike may also have risen from the essential nature of anatomy education, which is difficult to translate to an online format. The fact that consensus statement “I think that virtual specimens do not replace the physical presence of specimens” was strongly agreed with by all groups emphasizes the issues with online anatomy education.

In conclusion, on-line education will likely continue and further evolve beyond the pandemic^32^. This study will be useful in providing insights from the students’ perspectives. These granular findings indicate how students might differ on viewing and evaluating online courses, and this methodology can be used in redesigning and restructuring different components of an online course in higher education settings.

## Supplementary Information

Supplementary Tables.

## Data Availability

The datasets used in this study are not publicly available because of confidentiality, however, it can be made available by the corresponding author upon reasonable request.
