# Supplementary material for: A longitudinal Q-study to assess changes in students’ perceptions at the time of pandemic
Source: Sci Rep. 2023 May 30;13:8770. doi: 10.1038/s41598-023-36003-9 (PMC10228893; doi:10.1038/s41598-023-36003-9)
Supplement: Supplementary file 1 — Supplementary Tables. [file 41598_2023_36003_MOESM1_ESM.docx]

| Concourse | The initial set of statements collected from participant surveys, interviews and focus groups, review of prior concourses, literature review, etc. |
| --- | --- |
| Q-sample (Q-set) | A revised and representative list of statements from the concourse given to participants for data collection. This list is usually assembled by deleting the similar statements in the concourse and minimal revision of the statements for readability |
| Q-sort table | The table provided to participants for sorting statements within the Q-sample – this table has a quasi-normal distribution and should have as many cells as the number of statements in the Q-sample |
| Q-sort | A completed Q-sort table which includes a participant’s rank-ordered set of statements |
| Factor | A group of participants with similar viewpoints, opinions, perceptions, or preferences as presented by the participants’ Q-sorts. Factors are extracted using a by-person factor analysis on all Q-sorts |
| Factor loadings | Factor loadings represent the correlations between participants and factors |
| Factor score | Denotes level of agreement between statement and a factor |
| Distinguishing statement | A distinguishing statement for each factor is a statement that its factor score for the factor is significantly different from its factor scores on all the other factors |
| Consensus statements | A statement that all participants (loaded on all factors) agree with or disagree with at the same level. Indeed, there is no statistically significant difference between factor scores of the statement across factors |

**Supplementary Table S1.** Key terms used in Q-methodology

| **Statement # Statement** | | **Factor 1** | **Factor 2** | **Factor 3** |
| --- | --- | --- | --- | --- |
| 1 | I feel that the expectations for the peer teaches/presentations are unclear. | -1 (-3) | 0 (-2) | -1 (-1) |
| 2 | I believe working in a group for peer teaches/presentations helped me learn and apply communication skills. | 0 (-2) | -2 (-2) | 1 (-1) |
| 3 | Asynchronous lab modules were critical to my understanding of anatomy. | -4 (-4) | -1 (2) | 1 (-1) |
| 4 | I think lectures covered an appropriate amount of content. | -5 (-4) | -1 (2) | -1 (4) |
| 5 | I'm comfortable with the technology skills required for studying anatomy online. | -2 (2) | 3 (4) | 4 (3) |
| 6 | I find that there is not much distinction between synchronous labs and tutorials. | 1 (-2) | 2 (4) | 4 (2) |
| 7 | Watching lectures was a waste of my time. | -3 (-5) | -5 (-5) | -5 (-4) |
| 8 | Tutorials are useless to me. | -4 (-2) | 4 (0) | -5 (-2) |
| 9 | I found it difficult to keep up throughout the semester - it was super easy to fall behind. | 4 (4) | 3 (0) | 1 (0) |
| 10 | I think that virtual specimens do not replace the physical presence of specimens. | 2 (4) | 5 (5) | 4 (5) |
| 11 | The long answer worksheets are beneficial to my learning. | -2 (-3) | -4 (-3) | -1 (-2) |
| 12 | I think there should be transcripts for asynchronous lectures. | 4 (5) | 2 (1) | 2 (1) |
| 13 | The professors are helpful for my understanding of course content. | -2 (1) | 0 (1) | -2 (2) |
| 14 | I think there should be a standard set of slides/specimens that all groups will cover in synchronous labs and tutorials. | 1 (3) | 3 (3) | 2 (1) |
| 15 | I feel that the MCQ evaluations often require far more integration and application than we are taught in lecture, lab, and tutorial. | 2 (1) | -2 (-3) | -4 (-4) |
| 16 | I find the synchronous sessions to be a toxic environment because some students will try to show off. | -3 (-4) | -4 (-5) | -4 (-5) |
| 17 | There needs to be more consistency between the slides of the different lecturers. | 2 (1) | -1 (1) | 0 (-2) |
| 18 | I need more time to complete my MCQ exam. | 5 (0) | 0 (-4) | -3 (-3) |
| 19 | I feel that the "ask the faculty" sessions are a comfortable space to ask questions. | -1 (-3) | -3 (-1) | -1 (-1) |
| 20 | I believe having multiple professors from different areas of specialty is a strength of this course. | -4 (-1) | 1 (3) | 0 (2) |
| 21 | I believe the transition to online school has removed the opportunity to learn from and communicate with other students. | 1 (3) | 0 (2) | 0 (3) |
| 22 | I feel that the different components of the course (lecture, lab, and tutorial) complement each other well in a way that is effective to my learning. | -3 (0) | -2 (-1) | -2 (0) |
| 23 | I like that the asynchronous lectures allow me to stop, rewind, and listen to lectures multiple times. | 2 (4) | 4 (4) | 3 (4) |
| 24 | I have often found that the content in the lectures and the tutorials do not line up. | -1 (-2) | 1 (-3) | 1 (-4) |
| 25 | I feel like I am teaching myself. It is like paying tuition to watch YouTube videos. | 5 (3) | 1 (0) | -1 (1) |
| 26 | I would benefit from more faculty-made bellringer and short answer practice. | 3 (5) | 5 (5) | 5 (5) |
| 27 | I think that the lectures fostered connections between anatomy and physiology. | 0 (1) | 1 (3) | 2 (4) |
| 28 | I think the way in which we are evaluated does not fairly represent what the material covered. | 4 (1) | 2 (0) | -3 (-3) |
| 29 | I think the content is definitely useful and applicable to my future career. | 0 (2) | 2 (2) | 2 (0) |
| 30 | I think TA office hours are very helpful. | 0 (1) | -3(1) | 3 (0) |
| 31 | I prefer online learning compared to the in-person format. | -5 (-5) | -3 (-2) | -3 (-5) |
| 32 | Synchronous lab sessions were critical to my understanding of anatomy. | 1 (-1) | -5 (-4) | 1 (-1) |
| 33 | I learned about the systems that work together in a holistic approach, rather than about individual, specific anatomy. | -2 (0) | -1 (3) | 1 (3) |
| 34 | I think I would perform better on an in-person exam than an online exam. | -1 (0) | -1 (-1) | -2 (-2) |
| 35 | I think we were tested too much on small insignificant names and details instead of bigger ideas. | 3 (-1) | 1 (-1) | -3 (-2) |
| 36 | I feel that learning from virtual human prosections is a privilege. | -1 (-1) | 0 (2) | 0 (1) |
| 37 | I think the lab modules are easy to follow. | -3 (0) | 3 (2) | 3 (2) |
| 38 | I found the textbook was useful and supported what I learned in lecture. | -2 (-3) | -4 (-3) | 0 (0) |
| 39 | I think that the quality of teaching is worse than prior to the pandemic. | 3 (2) | -1 (-1) | 0 (0) |
| 40 | I think the Anatomy Department needs to create a more supportive and encouraging learning environment. | 2 (2) | 0 (0) | -2 (-1) |
| 41 | Watching in-person lecturers use their body to emphasize concepts really helps cement them in my brain. | 1 (2) | 2 (1) | 5 (3) |
| 42 | I think creating peer teaches/presentations was a useful way to learn and remember content. | 0 (0) | -3 (-2) | 2 (2) |
| 43 | I get confused because course information is on more than one platform. | 0 (-2) | -2 (-4) | -4 (-3) |
| 44 | I have difficulty understanding and practicing for the bellringer using the virtual specimens. | 3 (3) | 4 (0) | 3 (1) |

**Supplementary Table S2.** List of Q-sample statements used for data collection at midterm (fall 2020- Time 1) and at the end of term (winter 2021-Time 2) with their ranked factor scores for the 3 extracted factors at Time 1 (Time 2)
